# Supplementary material for: Incidence and predictors of mortality among road traffic accident victims admitted to hospitals at Hawassa city, Ethiopia
Source: PLoS One. 2024 May 29;19(5):e0296946. doi: 10.1371/journal.pone.0296946 (PMC11135675; doi:10.1371/journal.pone.0296946)
Supplement: Attachment — Submitted filename: Response to Reviewers.docx [file pone.0296946.s002.docx]

**Additional requirements that should addressed**

1. Please ensure that your manuscript meets PLOS ONE's style requirements, including those for file naming.

- I tried to follow PLOS ONE's style requirements

2. We noticed you have some minor occurrence of overlapping text with the following previous publication(s), which needs to be addressed:

- The document you have attached was done for fulfillment of Masters Degree in Hawassa University. It was my work attached for junior students in institutional repository but it was not manuscript since the whole document must attached there for the accomplishment of MPH IN EPIDEMIOLOGY.

3. We note that you have indicated that data from this study are available upon request. PLOS only allows data to be available upon request if there are legal or ethical restrictions on sharing data publicly.

- I have attached the anonymized version of data in a place where as supporting files

4. Please include your full ethics statement in the ‘Methods’ section of your manuscript file. In your statement, please include the full name of the IRB or ethics committee who approved or waived your study, as well as whether or not you obtained informed written or verbal consent. If consent was waived for your study, please include this information in your statement as well.

- We attached the details in the ethical consideration part
- The informed consent was waived because we used secondary data for our study, but we get permission from higher officials of institutions included in the study
- The name of IRB was Hawassa University College of Medicine and Health Sciences Institutional Review Board

**Comments to the author section (Reviewer #1)**

1. Is the manuscript technically sound, and do the data support the conclusions?

- Yes

2. Has the statistical analysis been performed appropriately and rigorously?

- Yes

3. Have the authors made all data underlying the findings in their manuscript fully available?

- Yes

4. Is the manuscript presented in an intelligible fashion and written in Standard English?

- Yes

5. "The World Health Organization's (WHO) 2023 report shows that 1.3 million people die and 20 to 50 million more are injured each year as a result of RTAs around the world. More than 90% of the world’s road traffic fatalities occur in low- and middle-income countries, even though these countries have a limited number of vehicles [4]"

- Changed to recent global data

6. Please clearly state inclusion criteria and exclusion criteria for the study population. Whether mono trauma is such as distal radius fracture part of this investigation?

- Our study outcome variable was occurrence of mortality among road traffic accident victims admitted to hospitals. Therefore; we were not differentiating between affected body parts since we followed all accident victims with in study period for event (death).
- We were also studied predictors of mortality (event) with in study period among road traffic accident victims. The affected body part might had significant impact on mortality of victims, that means those having had head injury could be more prone to death as compared to having had extremity injury though we included all victims of road traffic accident.

7. "WHO [13]" This reference is 22 years old. Please explained in great detail

- We used this reference for development of data collection tool, since it was additional to other literatures reviewed and no other guideline developed after it.

8. The advantages and disadvantages of this study design

- The advantage was being follow up study which stronger than cross-sectional/case-control studies
- The disadvantage was being retrospective using secondary data

9. "Important modifications were made" Which kind of modifications were made and why?
10. Modifications made were due to incompleteness data from secondary sources like socio-demographic factors; marital status, occupations, pre-hospital factors; management given and others after pretesting of data collection tools.
11. "398 road traffic accident victims" there is a discrepancy in the previous reported 403 included patients to this statement. Where are the 5 missing patients?

- The final sample size was 403, but the study conducted in 398 patient cards. the rest 5 patient cards were excluded due to incomplete data.

12. "The majority, 89.45% of the victims, had no comorbid conditions. (Table 1)" What kind of comorbid conditions where these? Are they contributing to the outcome? Such as blood-thinners are pre-existing severe illnesses?

- Comorbid conditions were pre-existing chronic diseases like diabetes mellitus, hypertension, HIV/AIDS, cancers of different origins and others which had significant effect on the outcome variable

13. "More than one-third (36.93%) of the victims have had poly-trauma; whereas 35.17% were injuries to multiple body regions". Please clearly state the difference here again, so the reader can follow.

- The first one was about occurrence of poly-trauma (35.17%); yes there was error in typing

14. "About 64.82% of the road traffic accident victims had to bleed"

- They were bleeding at the time of arrival to the hospital or prior to that after accident had been occurred.

15. Please rephrase the sentence.
16. "The most commonly performed management procedure was fixation (30.90%)"

- More than a quarter (30.90%) of victims had got fixation as a management procedure

17. To get a better understanding of the mortality rate, it is important to differentiate between the first surgical treatment of the patient from the external referring hospital and the receiving hospital.

For example, when were the interventions carried out? Since the majority of the patients were transferred from different hospitals, the need to be a distinction of when the surgical interventions were carried out.

- Really, this was very important concern for our study, but it was very difficult to get this information from secondary data for all victims. Therefore, it could be the limitation of our study.
- As it was follow-up study, we were interested to follow the victims after admission to discharge in the selected health facilities. So that it was impossible to follow outside the selected health facilities.

18. In the described mortality rate, what was the leading cause of death? It is described in great detail, that the GCS values and a high impact on mortality. However accompanying injuries and leading cause of death is not clear to me.

- As to our understanding, it was not the objective of this study and we tried to meet the objectives well.

19. "The current study revealed that the incidence of mortality among road traffic accident victims in the age group above 50 years was high, which is not consistent with studies undertaken in Yemen, Ghana, the D.R. Congo, and Ethiopia where the rate of death was higher among the age group below 50 years"[12, 20, 21, 25]
20. "Another possible reason might be that most of the study victims who arrive at these hospitals spent their time in another institution without getting adequate treatment."

- "Another possible reason might be most of the study victims who arrive at these hospitals spent their time in another institution without getting adequate treatment."
- According to our study, nearly three fourth (72.61%) of road traffic accident victims have had referred from another health facility which might cause delay without getting adequate management.

**Comments to the author section (Reviewer #1)**

1. Here median is sometimes presented with SEM (standard error of median), where IQR should be stated. Further some of SEM presented doesn’t make sense. Please reevaluate and correct where necessary.

- A total of 398 patients were followed for a range of 24 to 720 hours, and median follow-up time was **120 +/- 144 hours. Here, the median was 120 hours and IQR was 144 hours.**
- If any discrepancy in the data set, we can see it! Really we appreciate the critical observation of reviewer.

2. Could the authors please shortly explain the rationale for using patient hours of observation as the metric for conveying mortality rate?

- Our analysis plan was using survival analysis; so it needs time to event data set and the event was death.

3. I would recommend adding this data in table form to make it more easily understood.

- We omit it from table to avoid duplicate presentation

4. In the results section, the data from Table 6 should be presented.

- We think table 6 was not important here, really we thanks for detail observation

5. Further, in Table 1, please remove “having removal form” as this is redundant since referral status was also listed.

- It was ‘having referral form’; the first part or referral status was added if some of them might not have referral form.
